# A Manual of the Practice of Medicine

**Published:** 1893-09

**Authors:** 


*A Manual of the Practice of Medicine.* By A. A. STEVENS, M.D. Pp. 501. Philadelphia: W. B. SAUNDERS. 1893.

The preface informs us that this book was written at the request of many students, and is intended to be used as an outline, which shall be enlarged upon by diligent attendance upon lectures and critical observation at the bedside. As an outline we think it worthy of all commendation, and as a cram book for examinations it should be of service; but we have no sympathy with the student who would be content with such a manual.

On page 349, physical symptoms must be intended for psychical.

